# Supplementary figures and images for: Concurrent Evolution of Antiaging Gene Duplications and Cellular Phenotypes in Long-Lived Turtles
Source: Genome Biol Evol. 2021 Nov 18;13(12):evab244. doi: 10.1093/gbe/evab244 (PMC8688777; doi:10.1093/gbe/evab244)

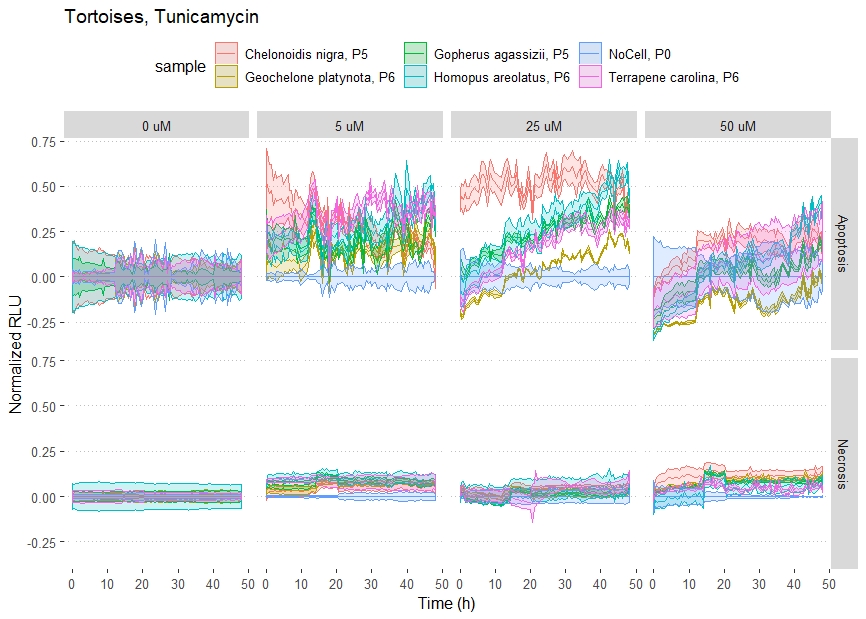

Supplement: evab244_Supplementary_Data [file evab244_supplementary_data.zip › Source data files and figure supplements/Figure 4 ΓÇô Figure Supplement 1. Tunicamycin Timecourse.jpg]

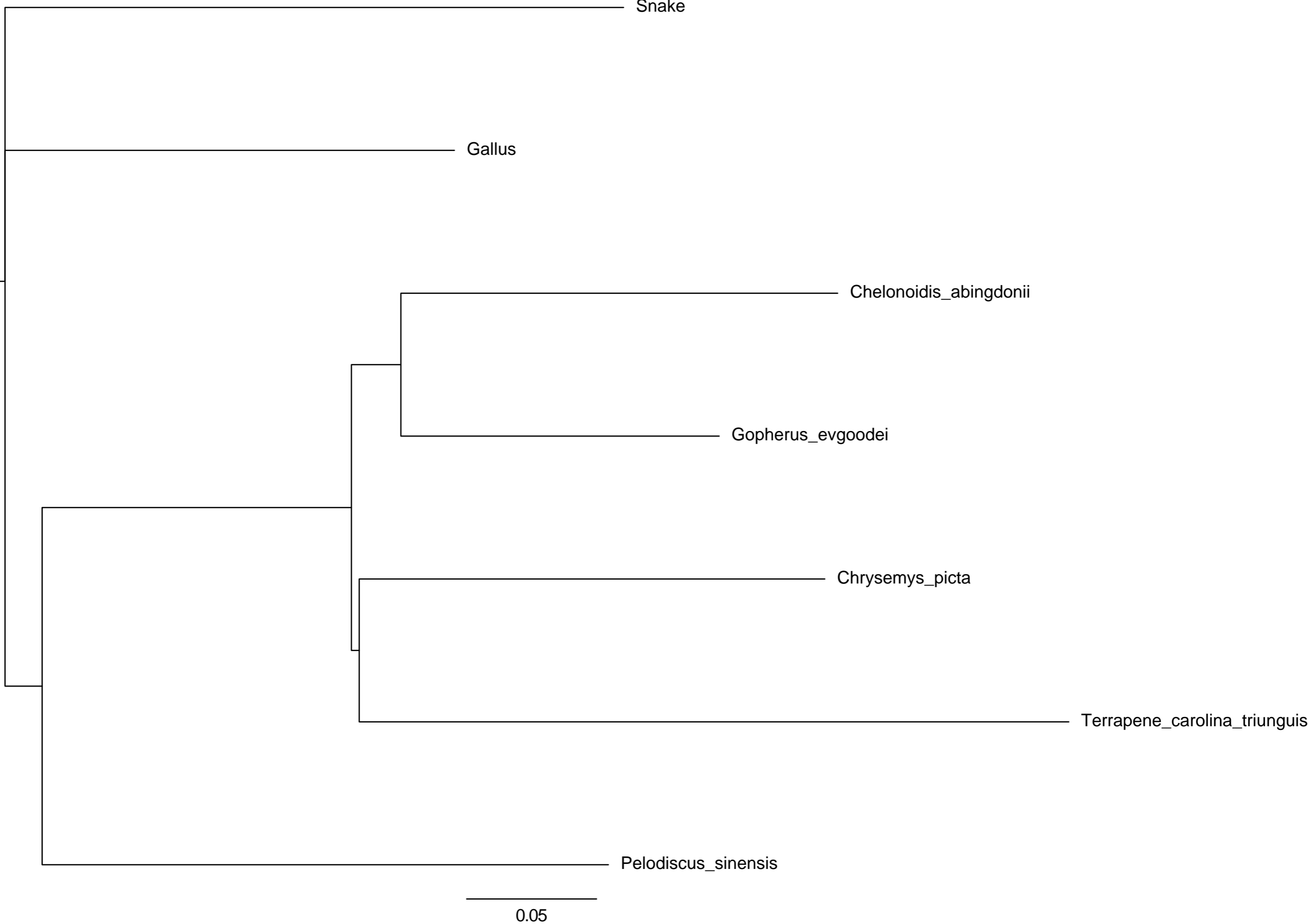

Supplement: evab244_Supplementary_Data [file evab244_supplementary_data.zip › Source data files and figure supplements/Figure 3 ΓÇô source data 1. Ancestral reconstruction of copy number changes/IQTREE/Constraint.tree.pdf]

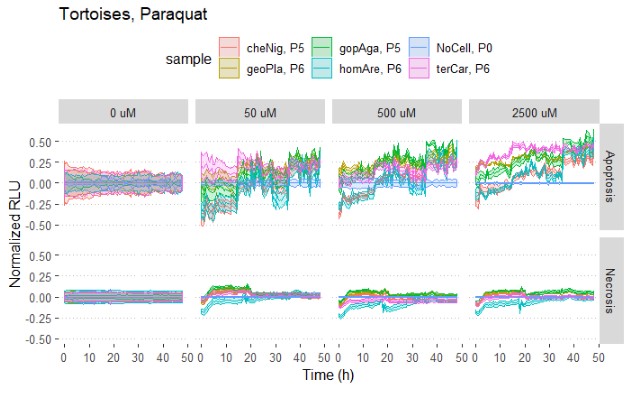

Supplement: evab244_Supplementary_Data [file evab244_supplementary_data.zip › Source data files and figure supplements/Figure 4 ΓÇô Figure Supplement 3. Paraquat Timecourse.jpg]

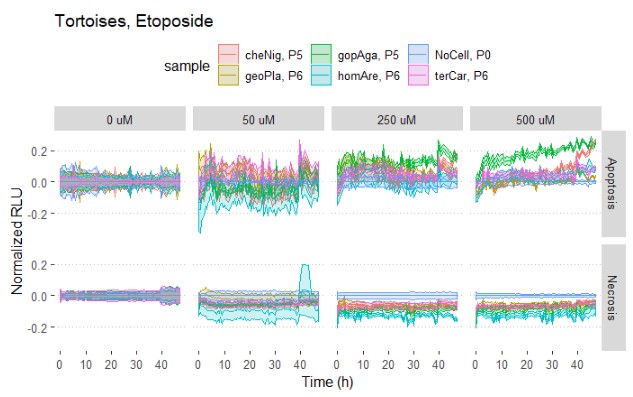

Supplement: evab244_Supplementary_Data [file evab244_supplementary_data.zip › Source data files and figure supplements/Figure 4 ΓÇô Figure Supplement 2. Etoposide Timecourse.jpg]

$\ln\text{Lifespan} \sim \ln\text{Size}$

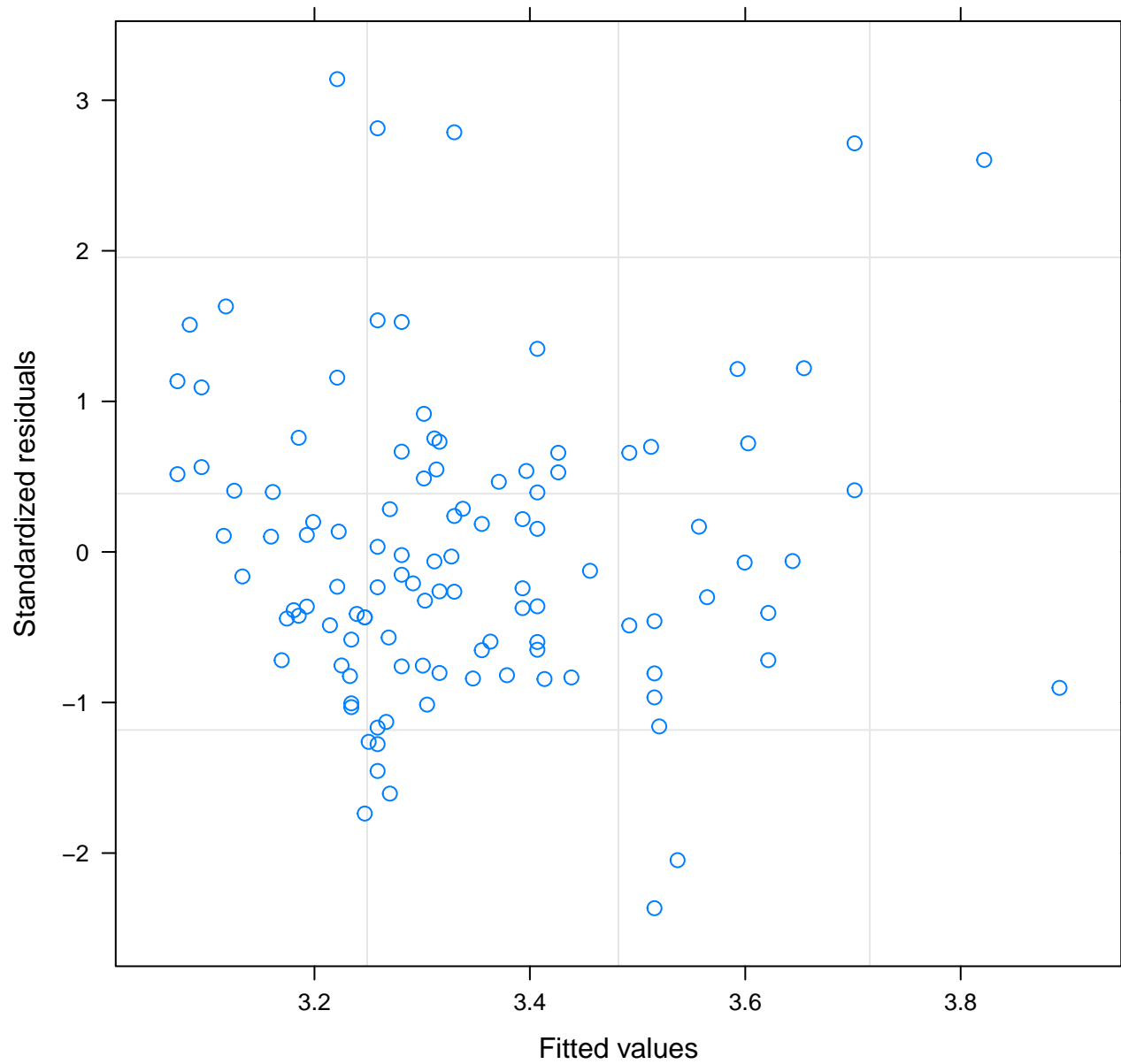

Supplement: evab244_Supplementary_Data [file evab244_supplementary_data.zip › Source data files and figure supplements/Figure 2 ΓÇô source data 1. Ancestral reconstructions of testudine body size, lifespan, and intrinsic cancer risk/RICR data/lifespan-PGLS-Testudines_noGuntheri_residualvsfitted.pdf]

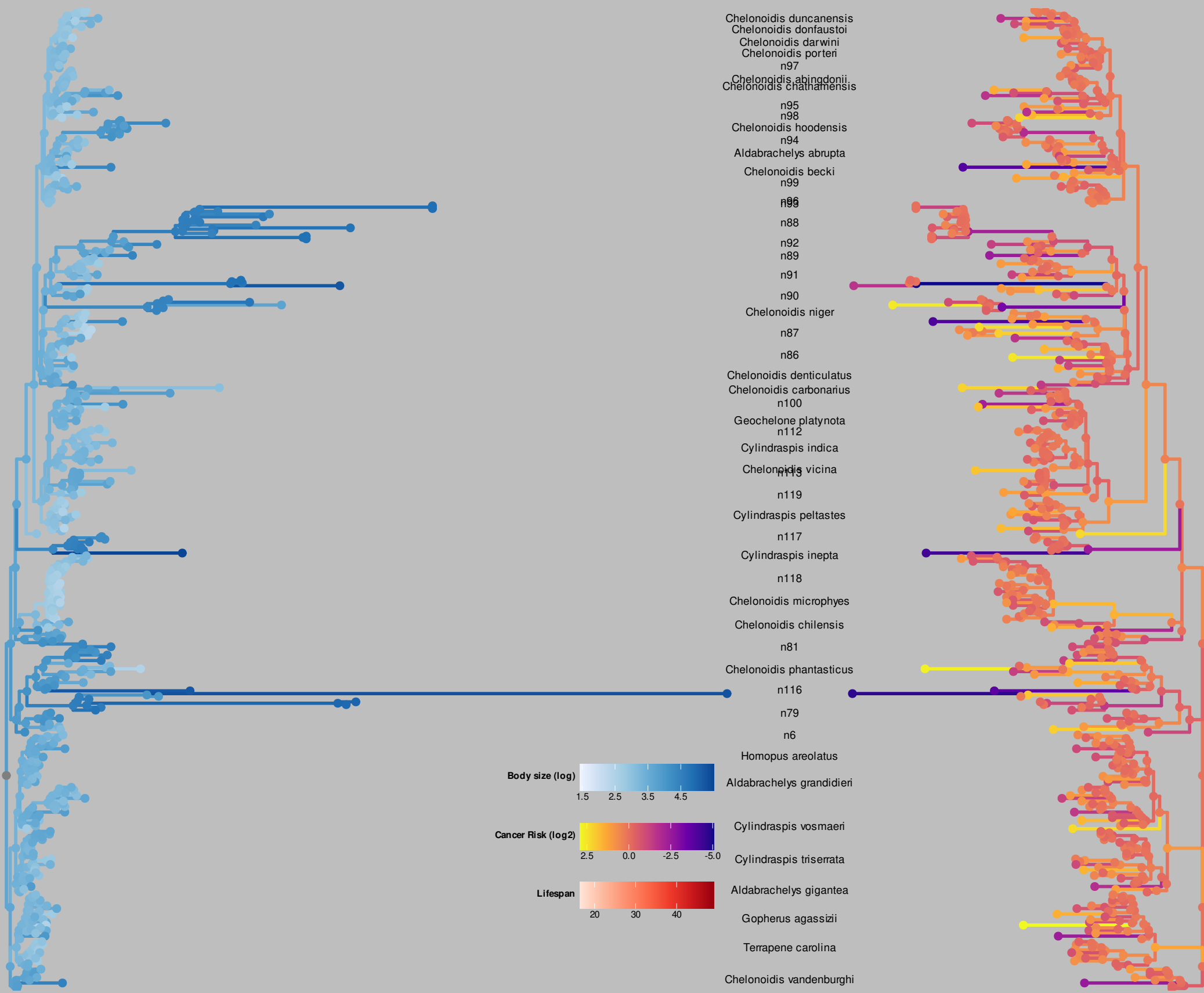

Supplement: evab244_Supplementary_Data [file evab244_supplementary_data.zip › Source data files and figure supplements/Figure 2 ΓÇô source data 1. Ancestral reconstructions of testudine body size, lifespan, and intrinsic cancer risk/RICR data/Testudines_noGuntheri-doubletree.pdf]

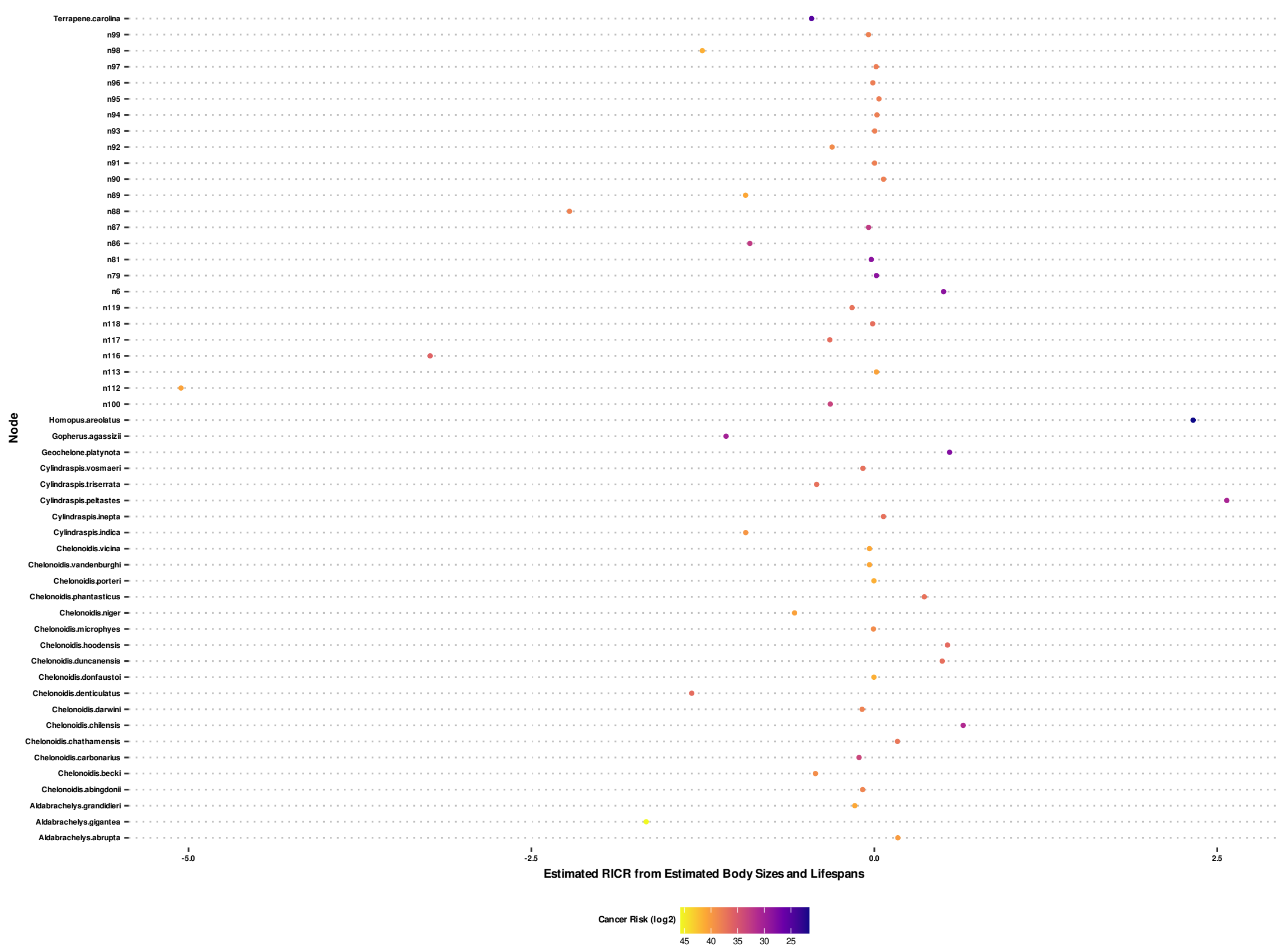

Supplement: evab244_Supplementary_Data [file evab244_supplementary_data.zip › Source data files and figure supplements/Figure 2 ΓÇô source data 1. Ancestral reconstructions of testudine body size, lifespan, and intrinsic cancer risk/RICR data/Testudines_noGuntheri-RICR.pdf]

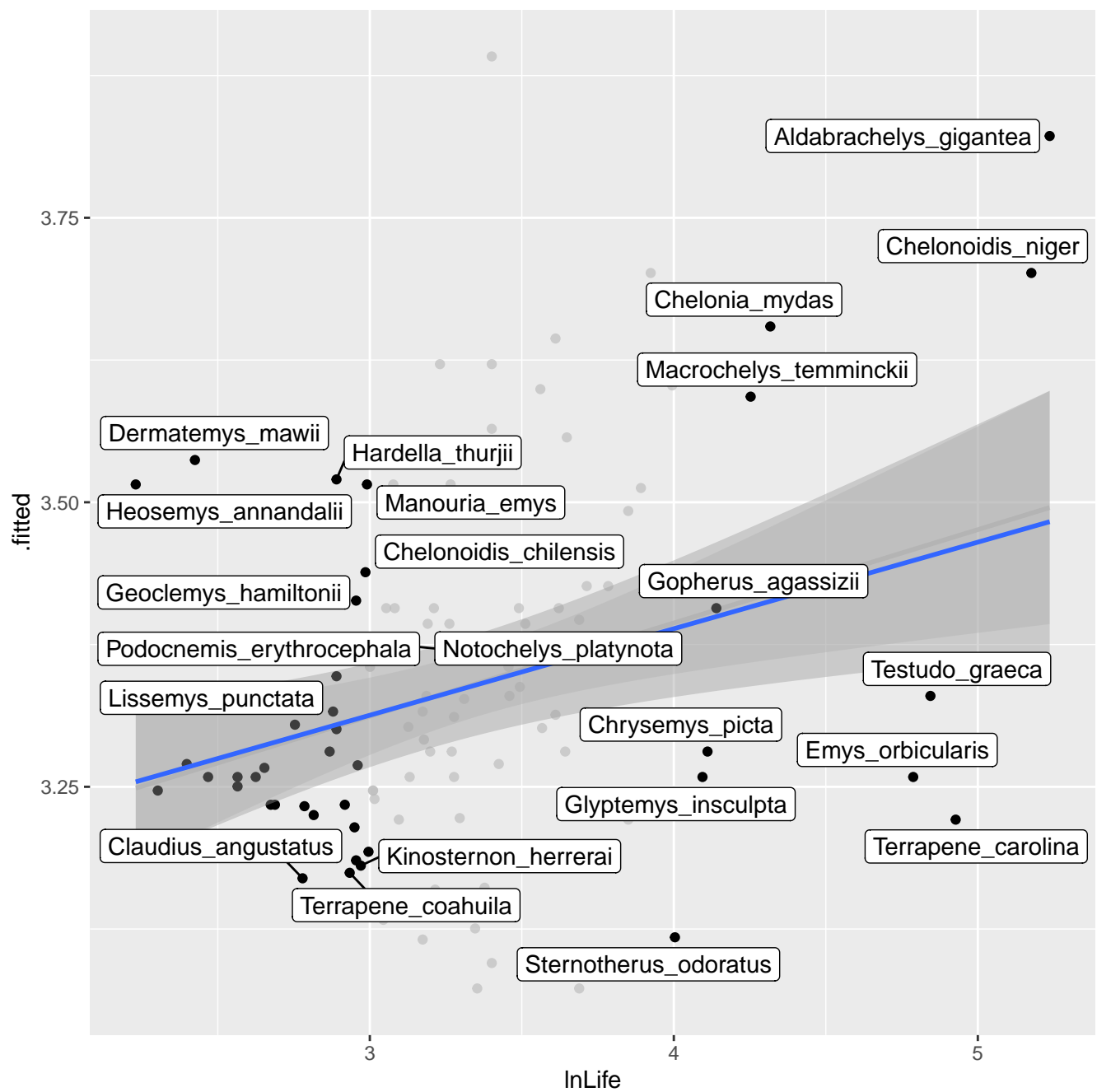

Supplement: evab244_Supplementary_Data [file evab244_supplementary_data.zip › Source data files and figure supplements/Figure 2 ΓÇô source data 1. Ancestral reconstructions of testudine body size, lifespan, and intrinsic cancer risk/RICR data/lifespan-PGLS-Testudines_noGuntheri_realvspred.pdf]

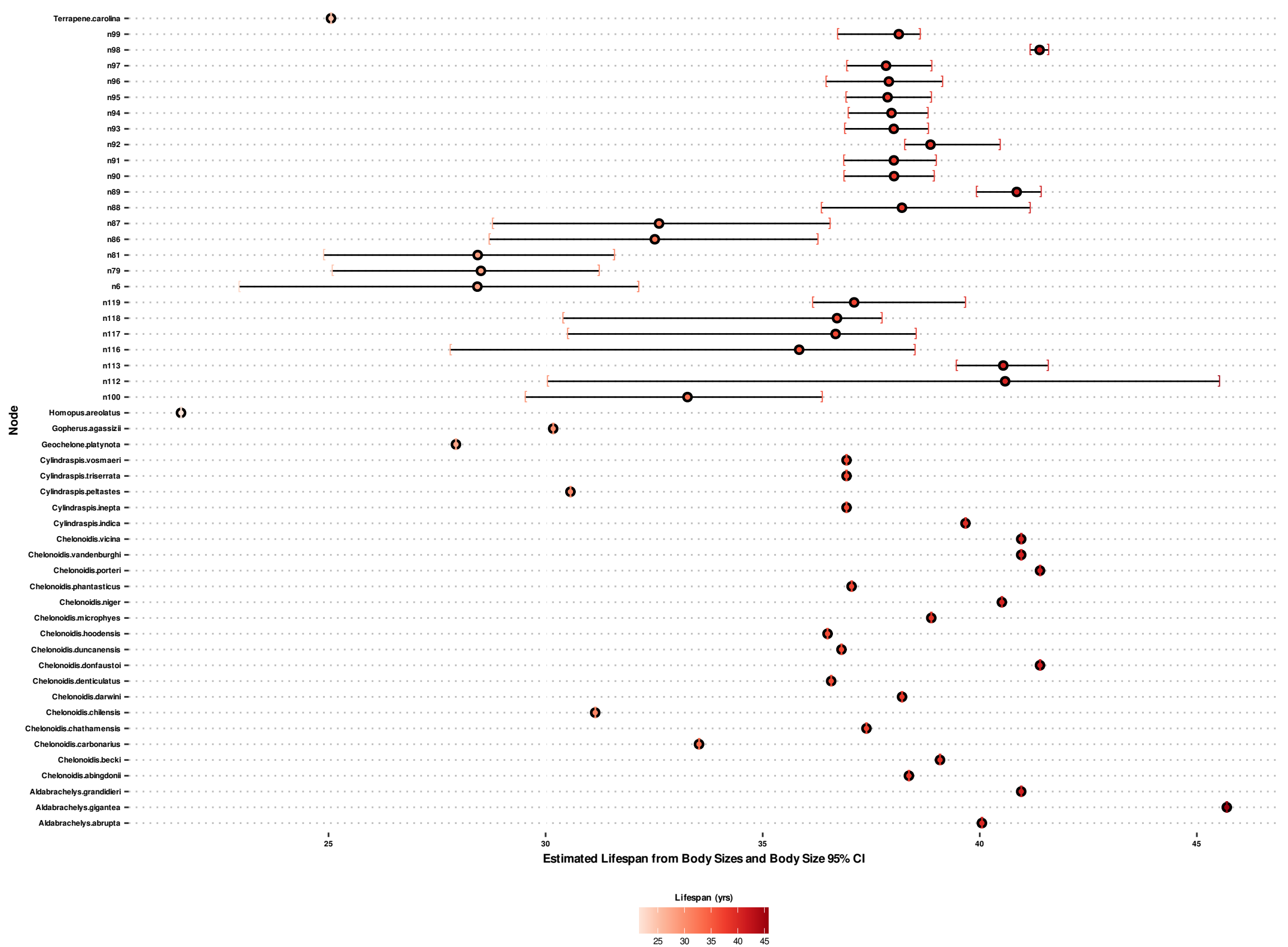

Supplement: evab244_Supplementary_Data [file evab244_supplementary_data.zip › Source data files and figure supplements/Figure 2 ΓÇô source data 1. Ancestral reconstructions of testudine body size, lifespan, and intrinsic cancer risk/RICR data/Testudines_noGuntheri-lifespan.pdf]

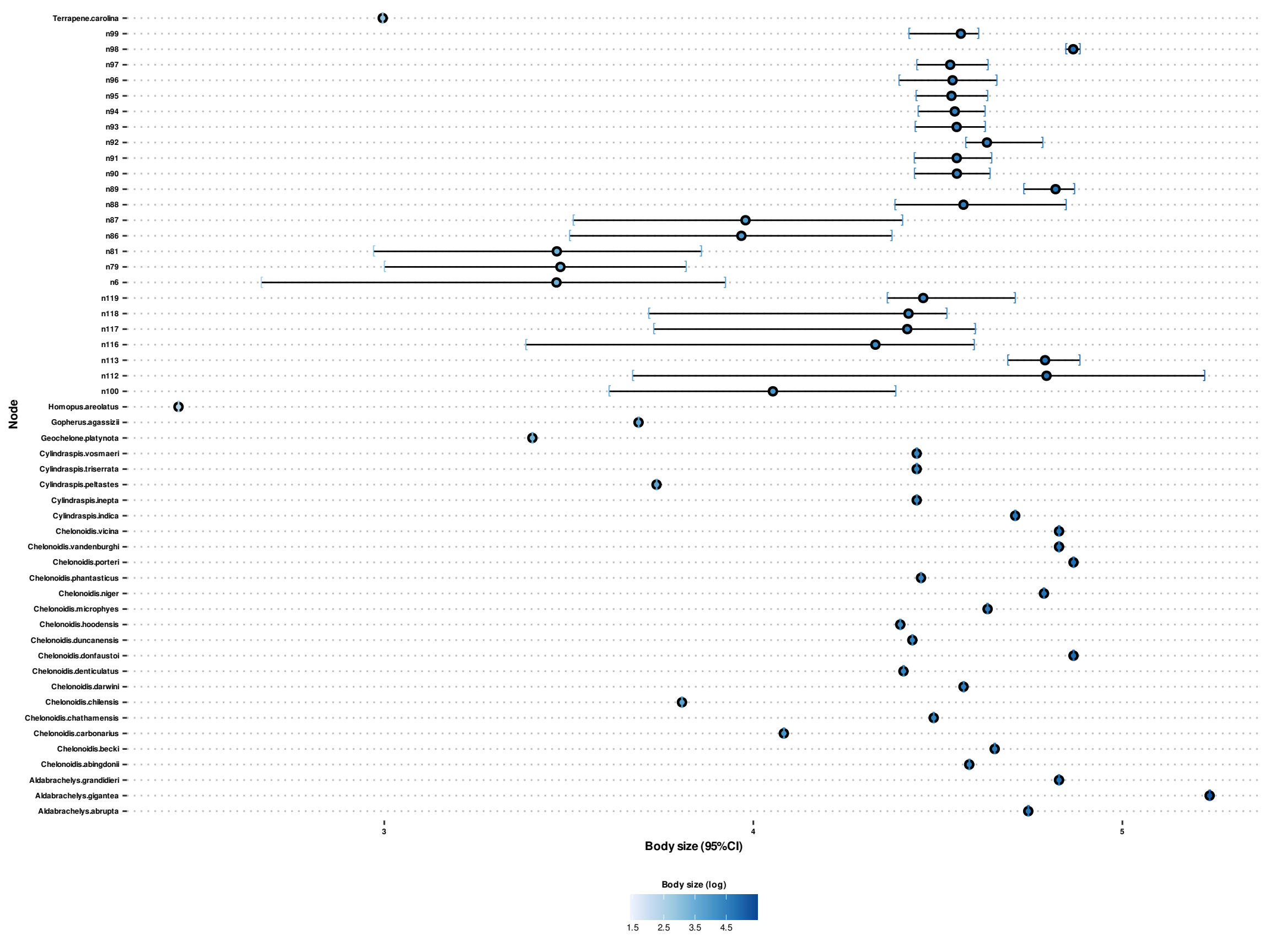

Supplement: evab244_Supplementary_Data [file evab244_supplementary_data.zip › Source data files and figure supplements/Figure 2 ΓÇô source data 1. Ancestral reconstructions of testudine body size, lifespan, and intrinsic cancer risk/RICR data/Testudines_noGuntheri-bodysize.pdf]
